# The complete mitochondrial genome sequence of *Desis martensi* (Araneae: Desidae)

**DOI:** 10.1080/23802359.2020.1839368

**Published:** 2020-12-24

**Authors:** Run-Biao Wu, Lu-Yu Wang, Zhi-Sheng Zhang

**Affiliations:** Key Laboratory of Eco-Environments in Three Gorges Reservoir Region (Ministry of Education), School of Life Sciences, Southwest University, Chongqing, China

**Keywords:** *Desis martensi*, mitochondrial genome adaptation, phylogenetic relationship

## Abstract

The complete mitochondrial genome sequence of *Desis martensi* (L. Koch, 1872) was reported. In this study, we sequenced, assembled and annotated the mitochondrial genome of *Desis martensi* using next-generation sequencing (NGS). The sequence was 14,662 base pairs (bp) in length and consisted of 37 mitochondrial genes (13 protein-coding genes, 22 transfer RNAs, two ribosomal RNA genes). The overall base composition of the genome showed slightly A + T bias, AT content (77.2%) higher than GC content (22.9%). The phylogenetic analyses based on 13 protein-coding genes indicated that the family Desidae belonged to the Retrolateral Tibial Apophysis (RTA) clade in Araneae.

The family Desidae (Pocock, 1895) comprises 60 genera and 296 species (World Spider Catalog 2020), but no completely mitochondrial genome of any species has been sequenced in this family. Here, the mitochondrial genome of *Desis martensi* (Araneae: Desidae) was sequenced and reported. *Desis martensi* is one of 15 species of the genus *Desis* (Walckenaer, 1837), which lives in the intertidal zone that the habitat is covered by water at high tide and exposed at low tide. Samples were collected at Dadonghai (N18°13.078″, E109°30.903″ Alt. 9 m) in Jiyang district, Sanya city, Hainan Province, China, on 22 July 2019. Before genomic DNA was extracted, samples (label number: HNSY-19-722) were put into 100% ethanol and stored at −20 °C in the Arachnological collection at School of Life Sciences, Southwest University, Chongqing, China.

The four legs of spider were extracted using tweezers, and the whole genome DNA was extracted with the DNeasy Blood and Tissue Kit (Qiagen, Valencia, CA). DNA concentration was quantified using TBS-380 Fluorometer (PROMEGA, Madison, WI), total DNA mass 1.449 μg was used for sequencing using Illumina NovaSeq platform with the insert-size 400 bp paired-end (PE) at 2 Gb depth at Nanjing Yangzi Science and Technology Innovation Center, Nanjing, China. First, high-quality clean data were obtaining from raw sequencing data through three software: FastQC v0.11.7 (Andrews 2010) for quality control, AdapterRemoval v2.2.2 (Schubert et al. 2016) for adapter removing and SOAPec v2.03 (Luo et al. 2012) using the parameter Kmer 17 for quality correction on all reads. Second, clean data were assembled using NOVOPlasty v4.1 (Dierckxsens et al. 2017) with the parameter Kmer 39, annotated using MitoZ v2.4 (Meng et al. 2019) (for protein-coding genes) and MITOS WebServer (Bernt et al. 2013) (for tRNA and rRNA) with default parameters. Finally, we submitted the mitochondrial sequence to the NCBI database and got an accession number (MT982364).

The complete mitochondrial genome of *Desis martensi* is 14,662 bp in length, which consist 37 genes, including 13 protein-coding genes (PCGs), 22 tRNAs and two rRNAs. The overall base composition of the genome is 34.4% for A, 7.9% for C, 15% for G, and 42.8% for T, the higher value of A + T content (77.2%) compare to G + C content (22.9%), indicating there was slight A + T bias in *Desis martensi*. Among the 13 protein-coding genes, NADH dehydrogenase subunit 1 (ND1), NADH dehydrogenase subunit 4 (ND4), NADH dehydrogenase subunit 4 L (ND4L) and NADH dehydrogenase subunit 5 (ND5) were encoded in the Light (L) strand, and the remaining nine genes were encoded in the Heavy (H) strand. Eight tRNAs (trnY, trnC, trnL2, trnF, trnH, trnP, trnV, trnQ) were encoded located in the L-strand, and the other tRNAs were located in the H-strand. Both rrnL and rrnS were in L-strand. In 13 protein-coding genes, six (COX1, ND2, ND3, ND5, ND4, ND6) used ATA as start codon, two (COX2, COX3) used with TTG as start codon, four (ATP8, CYTB, ND1, ND4L) used ATT as start codon, one (ATP6) used ATG as start codons. Five (ATP6, COX2, ND3, ND5, ND6) ended with TAA as stop codon, three (ATP8, COX3, ND1) ended with TAG as stop codon, five (COX1, CYTB, ND2, ND4, ND4L) ended with T as stop codon.

One new and 17 published mitochondrial genome sequences downloaded from GenBank including *Trichonephila clavata* (Araneidae) as an outgroup were used for construction of phylogenetic tree. Subsequently, 13 protein-codon genes of each species sequence were extracted, aligned and concatenated using the software PhyloSuite v1.2.1 (Zhang et al. 2020). After that, the best select partitioning schemes and models of evolution were conducted with PartitionFinder2 (Lanfear et al. 2017), Bayesian inference tree was carried out with MrBayes v3.2.7 (Ronquist and Huelsenbeck 2003) using the parameters “ngen = 2,000,000 printfreq = 1000 samplefreq = 1000 nchains = 4 nruns = 2”. Finally, evolutionary tree was displayed with FigTree (Rambaut 2014). *Desis martensi* was clustered into RTA clade (Figure 1), the result showed that Desidae was one of the families in RTA clade.

**Figure 1.**
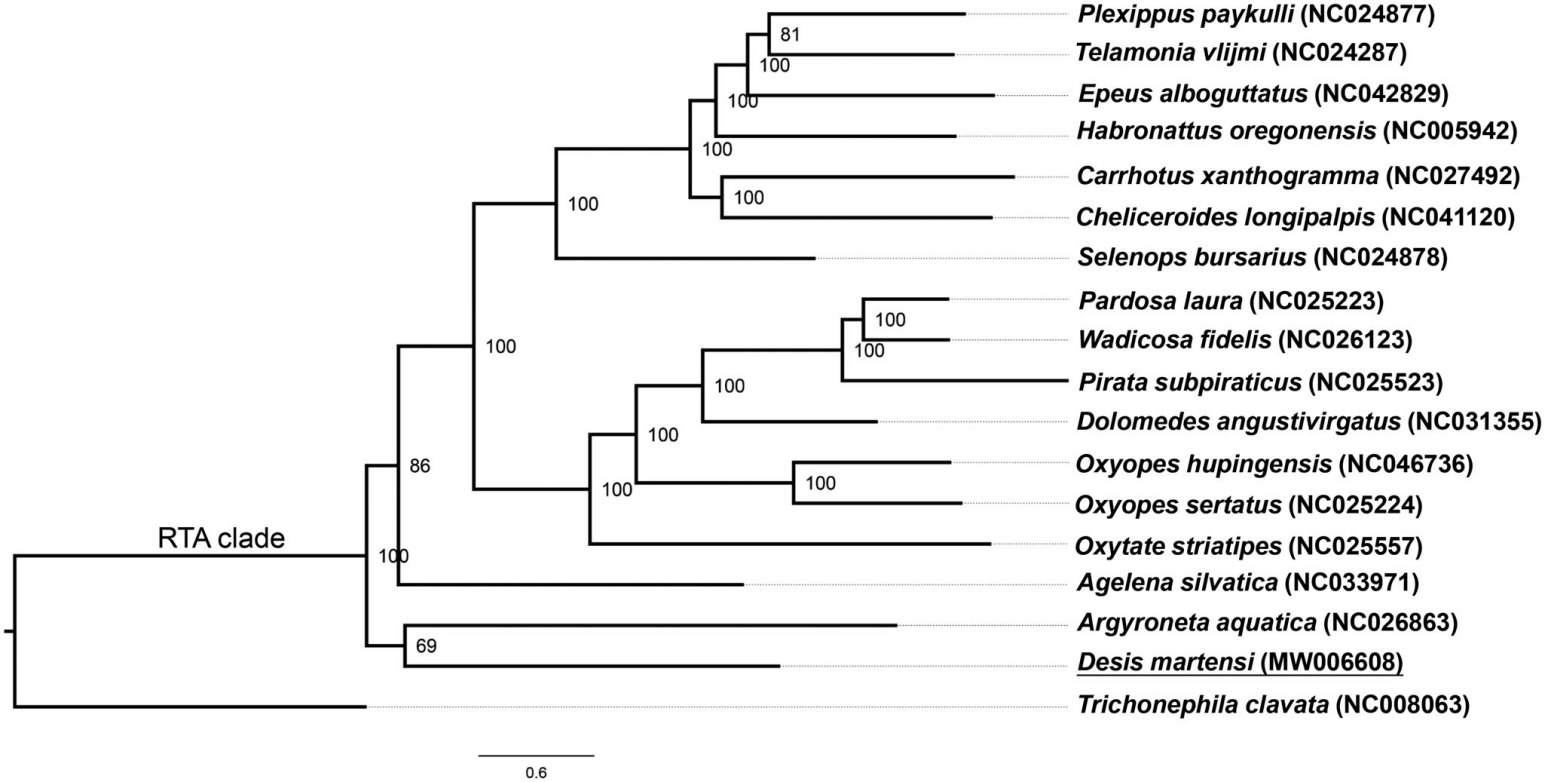
Phylogenetic relationships among 18 spiders based on 13 protein coding genes from mitochondrial genome, and numbers at nodes indicate Bayesian posterior probability values.

## Data Availability

The raw data for *Desis martensi* has been deposited in the NCBI SRA: BioProject ID PRJNA663105 at https://dataview.ncbi.nlm.nih.gov/object/SRR12639004, accession number (MW006608) in GenBank at https://www.ncbi.nlm.nih.gov/nucleotide/MT982364. The mitochondrial sequences of *Pirata_subpiraticus* (NC025523)*, Oxytate_striatipes* (NC025557), *Wadicosa fidelis* (NC026123), *Argyroneta aquatica* (NC026863), *Carrhotus xanthogramma* (NC027492), *Dolomedes angustivirgatus* (NC031355), *Agelena silvatica* (NC033971), *Cheliceroides longipalpis* (NC041120), *Epeus alboguttatus* (NC042829), *Oxyopes hupingensis* (NC046736), *Trichonephila clavata* (NC008063), *Habronattus oregonensis* (NC005942), *Telamonia vlijmi* (NC024287), *Plexippus paykulli* (NC024877), *Selenops bursarius* (NC024878), *Pardosa laura* (NC025223), and *Oxyopes sertatus* (NC025224) are openly available in GenBank at https://www.ncbi.nlm.nih.gov/nucleotide/.
